# Meiotic behaviour and its implication on species inter-relationship in the genus *Curcuma* (Linnaeus, 1753) (Zingiberaceae)

**DOI:** 10.3897/CompCytogen.v11i4.14726

**Published:** 2017-10-24

**Authors:** Judith Mary Lamo, Satyawada Rama Rao

**Affiliations:** 1 Plant Biotechnology Laboratory, North-Eastern Hill University, Shillong-793022, Meghalaya, India

**Keywords:** Polyploidy, amphidiploid, inter-specific crosses, diversification

## Abstract

In this paper, detailed meiotic analysis was investigated in seven species of *Curcuma* (Linnaeus, 1753) which can contribute significantly to our understanding about species inter-relationship, speciation and evolution. The species were divided into two groups viz., Group I having 2n = 42 (*C. comosa* Roxburgh, 1810, *C. haritha* Mangaly & M.Sabu, 1993, *C. mangga* Valeton & Zijp, 1917, and *C. motana* Roxburgh, 1800) and Group II with 2n = 63 (*C. caesia* Roxburgh, 1810, *C. longa* Linnaeus, 1753 and *C. sylvatica* Valeton, 1918). Both groups display varying degree of chromosome associations. Group I species showed the prevalence of bivalents, however occasional quadrivalents besides univalents were also encountered. About 48% of the PMCs analyzed in *C. mangga* showed 21 bivalents (II) meiotic configurations, 32% in *C. comosa* and 16% in *C. haritha*. Group II species as expected showed the presence of trivalents besides bivalents, univalents and quadrivalents. About 32% of the PMCs analyzed at MI in *C. sylvatica* showed 21 trivalents (III) meiotic configurations, 24% in *C. longa* and 8% in *C. caesia*. Overall, low frequency of multivalent associations as compared to bivalents indicates that *Curcuma* is an allopolyploid complex. Moreover, x = 21 is too high a basic number, therefore, we suggest that the genus *Curcuma* has evolved by hybridization of species with different chromosome numbers of 2n = 24 and 18, resulting in a dibasic amphidiploid species.

## Introduction

The genus *Curcuma* Linn. belonging to the tribe Zingibereae of the family Zingiberaceae consists of about 120 species and is pan-tropical in distribution (Kress et al. 2002, Škorničková et al. 2007, Záveská et al. 2012). It contains many taxa with multifaceted uses and quite a few species of *Curcuma* (e.g. *C. amada*, *C. caesia, C. longa*, etc.) are used as spice, dye, medicine, cosmetics, ornamental and as a source for starch (Sasikumar 2005, Velayudhan 2012).

*Curcuma*, a rhizomatous, perennial and herbaceous group of plant displays a great degree of diversity in ploidy levels which is evident from earlier cytogenetical studies wherein various chromosome numbers of 2n = 22, 42, 63, 77, 105, etc., have been reported. Moreover, continuous dispute concerning the basic chromosome number in *Curcuma* (x = 7, 8, 16 and 21) has been highlighted in early cytological studies of Raghavan and Venkatasubban (1943), Sharma and Bhattacharya (1959), Ramachandran (1961), Islam (2004), Škorničková et al. (2007). Whilst a lot of information on the somatic chromosome number is available for the genus *Curcuma*, essential information about the homology among the chromosome complements and level of polyploidy has yet to be investigated.

Meiosis, a highly conserved and specialized process in eukaryotes, not only generates genetic variability but also ensures gamete viability and constancy of ploidy levels (Pagliarini 2000, Hamant et al. 2006, Kumar and Singhal 2011, Brownfield and Köhler 2011). However, disruption of meiosis as well as pre- and post- meiotic events can have a severe effect on the genetic stability and viability of the gametes (Brownfield and Köhler 2011). Moreover, the degree of association and behaviour of chromosome pairing, chiasma distribution and its frequencies, disjunction of chromosomes in anaphase I/II can also provide significant insight on speciation and structural details of genomic organization and species inter-relationships (Sharma et al. 2011). Chromosome pairing, an important feature of meiosis, has often been used to infer genome relationship in hybrids and polyploid species (Grandont et al. 2013). Such studies might also contribute to the better understanding of cytological evolution of species which can be utilized for future genetic improvement and conservation of the genetic resources (Kumar and Singhal 2011). However, detailed studies on male meiosis are very much limited in the genus *Curcuma* except for a few reports of Ramachandran (1961), Nambiar (1979) and Puangpairote et al. (2016). The possible reason may be due to rare flowering of the plants under non-optimal environment and factors like inherent difficulty in obtaining good analyzable cytological preparations, small chromosome size and stainability (Puangpairote et al. 2016).

In this context, seven species of *Curcuma*: *C. comosa* Roxburgh, 1810, *C. haritha* Mangaly & M.Sabu, 1993, *C. mangga* Valeton & Zijp, 1917, *C. montana* Roxburgh, 1800, *C. caesia* Roxburgh, 1810, *C. longa* Linnaeus, 1753 and *C. sylvatica* Valeton, 1918, were taken up for the present investigation for analysis of meiotic pairing behaviour in order to find evidence on species inter-relationship, speciation and evolution. From our previous investigations on chromosome count, the somatic chromosome number in *C. comosa, C. haritha, C. mangga* and *C. montana* was observed to be 2n = 42 while 2n = 63 was recorded in *C. caesia, C. longa* and *C. sylvatica* (Lamo and Rao 2014, 2017).

## Material and methods

For the present investigation, *Curcuma* germplasm along with their specimen voucher numbers were obtained from Indian Institute of Spices Research, Kozhikode. Flower buds were obtained from the plants growing in polyhouse conditions at the Department of Biotechnology and Bioinformatics, North-Eastern Hill University, Shillong.

Flower buds of appropriate size were fixed in freshly prepared Carnoy’s solution (1:3 glacial acetic acid: 95% ethanol) for 4 days at room temperature and stored in 70% ethanol at 4ºC. Anthers were squashed in 2% aceto-carmine solution and in some cases ferric chloride solution was used as mordant. The slides were examined and photographed using Leica DM 4000 B microscope attached to Leica CCD camera at ×1000 magnification. For meiotic analysis each preparation was determined by microscopy as well as photomicrographs. On an average 25 PMCs/species were used for detailed analysis at diplotene, diakinesis and/or metaphase I.

The terminalization coefficient was calculated using the following formula:

**Figure:**
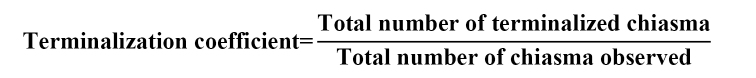


## Results

### Group I (2n = 42)

#### *C. comosa*

**Figure 1.**
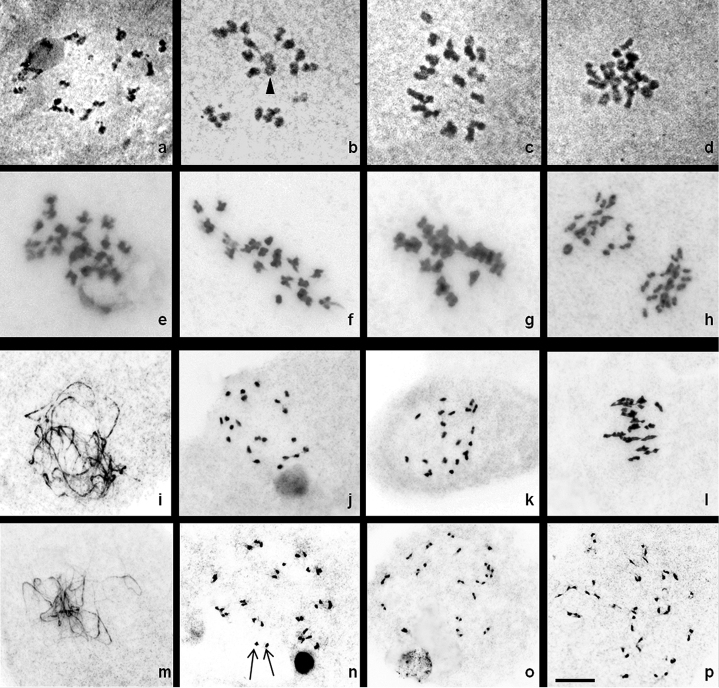
Male meiosis in group I. **a–d** *C. comosa*: **a** diplotene **b** diakinesis **c–d** metaphase I **e–h** *C. haritha*: **e** diakinesis **f** metaphase I **g–h** anaphase I **i–l** *C. mangga*: **i** pachytene **j** diplotene **k** diakinesis **l** metaphase I **m–p** *C. montana*: **m** pachytene **n** diplotene, **o–p** diakinesis; arrowhead showing multivalent and arrows showing univalents.Bar = 10 µm.

Chromosome associations at diplotene, diakinesis and metaphase I (MI) were characterised by both bivalents and univalents besides quadrivalents (Fig. 1a–d). About 32% of the PMCs were characterised by 21 bivalents (21II). Bivalents ranged from 13–21 with a mean value of 18.24. The bivalents showed both ring and rod association which ranges from 2–13 and 7–15 with a mean value of 7.44 and 10.80 respectively (Table 1). Quadrivalents ranged from 0 to 2 with a mean value of 0.68, whereas univalents ranged from 0–8 with a mean value of 2.80. No trivalent associations were encountered in any of the PMCs analysed. The total number of chiasmata observed was 796 out of which 619 were terminalized and 177 were unterminalized resulting in a terminalization coefficient of 0.78.

**Table 1.** Mean number and range of associations at diplotene/diakinesis/metaphase-I in Curcuma species.

Species	IISR Voucher No.	Chromosome associations																	
		Bivalent									Univalent			Trivalent			Quadrivalent		
		No.	Mean ±SD	Range	Ring			Rod			No.	Mean ± SD	Range	No.	Mean ± SD	Range	No.	Mean ± SD	Range
					No.	Mean ±SD	Range	No.	Mean ±SD	Range									
*C. comosa*	644	456	18.24 ±2.31	13–21	186	7.44 ±3.44	2–13	270	10.80 ±2.25	7–15	70	2.80 ±2.58	0–8	–	–	–	17	0.68 ±0.63	0–2
*C. haritha*	1136	395	15.80 ±2.27	13–21	80	3.20 ±1.50	0–6	315	12.64 ±3.1	11–12	208	8.32 ±3.90	0–16	–	–	–	13	0.52 ±0.59	0–2
*C. mangga*	1049	487	19.48 ±1.56	17–21	171	6.84 ±2.27	2–11	316	12.64 ±2.10	9–15	16	0.64 ±0.95	0–2	–	–	–	15	0.64 ±0.95	0–2
*C. montana*	649	421	16.84 ±1.91	12–20	174	6.96 ±1.97	4–12	247	9.86 ±2.60	6–15	96	3.84 ±2.60	0–12	–	–	–	28	1.12 ±0.93	0–3
*C. caesia*	751	361	14.44 ±7.80	0–24	102	4.08 ±2.38	0–8	259	10.36 ±6.16	4–20	70	2.90 ±2.45	0–8	213	8.52 ±6.73	0–21	36	1.44 ±1.22	0–3
*C. longa*	Pratibha	304	12.16 ±8.84	0–24	178	7.12 ±5.43	0–17	126	5.04 ±3.89	0–13	105	4.20 ±3.77	0–14	246	9.84 ±7.44	1–21	31	1.24 ±0.88	0–2
*C. sylvatica*	526	424	16.96 ±12.25	0–29	119	4.76 ±4.01	0–11	305	12.20 ±9.21	0–24	33	1.32 ±1.81	0–8	213	8.52 ±8.85	0–21	14	0.56 ±0.87	0–3

#### *C. haritha*

About 16% of the PMCs were characterised by the formation of 21II, while the remaining PMCs were characterised by both bivalent and multivalent associations besides univalents (Fig. 1e–h). The number of bivalents ranged from 13–21 with a mean value of 15.80 (Table 1). The ring bivalent ranges from 0–6 with a mean value of 3.20 and rod bivalents ranged from 11–12 with a mean value of 12.64. Quadrivalent associations ranged from 0 to 2 with a mean value of 0.52 and the total number of univalents was 208 with a mean value of 8.32. No trivalent associations were encountered in any of the PMCs analysed. The total number of chiasmata observed was 557 with a mean value of 22.28 (Table 2). The total number of terminalized chiasmata was 472 and unterminalized chiasmata were 85 yielding a terminalization coefficient of 0.85. About 72.73% and 27.27% of the PMCs analyzed showed 21:21 and 24:18 chromosome distributions at AI respectively.

**Table 2.** Mean number and range of chiasma, terminalization coefficient and percentage of pollen stainability in *Curcuma* species.

**Species**	**No of cells analysed**	**Chiasma**					**Terminalization coefficient**
		**Total**	**Mean ± SD**	**Range**	**Terminalized ± SD**	**Unterminalized ± SD**	
*C. comosa*	25	796	10.80±2.5	15–32	24.76±5.79	7.08±1.91	0.78
*C. haritha*	25	557	22.28±3.2.7	17–30	18.88±2.15	3.40±2.24	0.85
*C. mangga*	25	726	29.04±4.22	25–37	22.60±4.41	6.44±1.64	0.78
*C. montana*	25	718	28.72 ±3.61	28–40	23.72±5.56	5.92±2.38	0.82
*C. caesia*	20	1023	51.15±6.22	45–69	39.05±6.91	12.10±4.67	0.76
*C. longa*	25	676	27.04±19.62	0–49	19.76±14.68	7.28±5.34	0.73
*C. sylvatica*	28	1365	48.75 ±9.89	36–61	35.82±7.49	12.93±6.89	0.74

#### *C. mangga*

About 48% of the PMCs analysed showed 21II, while the rest showed a mix of both bivalent and multivalent associations besides univalents (Fig. 1i–l). The number of bivalents ranged from 17–21 with a mean value of 19.48 (Table 1). The ring bivalent ranged from 2–11 with a mean value of 6.84 and rod bivalents ranged from 9–15 with a mean value of 12.64. Quadrivalent associations observed ranged from 0–2 with a mean value of 0.64. Total number of univalents recorded was 16 with a mean value of 0.64. No trivalent associations were encountered in any of the cells analysed. The total number of chiasmata observed was 726 with a mean value of 29.04 ranging from 25–37 (Table 2). About 565 chiasmata were terminalized and 161 were unterminalized yielding a terminalization coefficient of 0.78.

#### *C. montana*

Detailed analysis at diplotene, diakinesis and metaphase showed that bivalents ranged from 12–20 with a mean value of 16.84 (Table 1; Fig. 1m–p). The number of ring bivalents ranged from 4–12 with a mean value of 6.96 and rod bivalents ranged from 6–15 with a mean value of 9.86. Quadrivalents ranged from 0 to 3 with a mean value of 1.12. Univalent lie in close proximity to each other and the total number of univalent recorded was 96 with a mean value of 3.84. No trivalent associations were encountered in any of the cells analysed. The total number of chiasmata observed was 718 with a mean value of 28.72 (Table 2). The total number of terminalized and unterminalized chiasmata was 593 and 148 respectively. Terminalization coefficient of 0.82 was being recorded.

### Group II (2n = 63)

#### *C. caesia*

**Figure 2.**
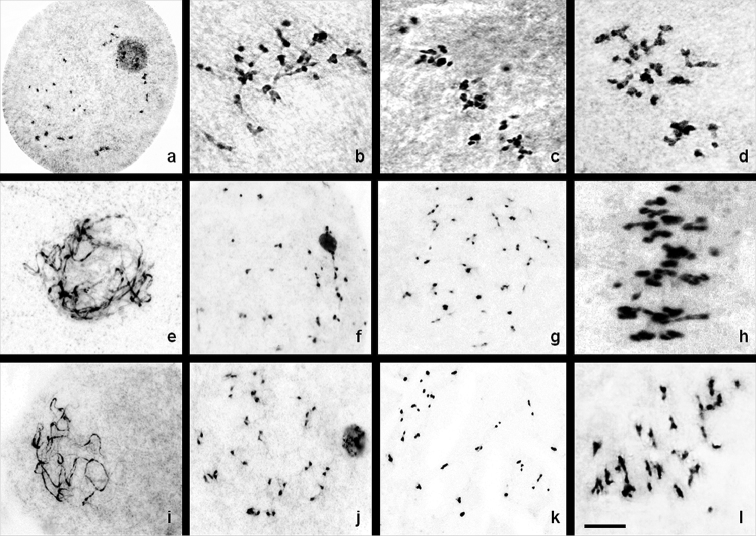
Male meiosis in Group II. **a–d** *C. caesia*: **a** diplotene **b** diakinesis **c–d** metaphase I **e–h** *C. longa*: **e** pachytene **f** diplotene **g** diakinesis **h** metaphase I **i–l** *C. sylvatica*: **i** pachytene **j** diplotene **k** diakinesis **l** metaphase I. Bar = 10 µm.

About 8% of the PMCs analysed showed trivalent associations (21III) while the rest showed both bivalent and multivalent associations along with univalents (Fig. 2a–d). The number of bivalents ranged from 0–24 with a mean value of 14.44 (Table 1). The ring bivalent ranges from 0–8 with a mean value of 4.08 and rod bivalents ranged from 4–20 with a mean value of 10.36. Trivalents ranges from 0–21 with a mean value of 8.52 while quadrivalents ranged from 0 to 3 with a mean value of 1.44. The total number of univalent recorded was 70. The total number of chiasmata observed was 1023 with a mean value of 51.15 (Table 2). Out of the 1023 chiasmata observed, 781 were terminalized and 242 were unterminalized yielding a termininalization coefficient of 0.76.

#### *C. longa*

About 24% of the PMCs analysed showed trivalent associations (21III) and the rest showed the occurrence of both bivalents and multivalents (trivalent and quadrivalent) associations along with univalents (Fig. 2e–h). The number of bivalents ranged from 0–24 with a mean value of 12.16 (Table 1). The ring bivalent ranges from 0–17 with a mean value of 7.12 and rod bivalents ranged from 0–13 with a mean value of 5.04. Trivalents ranged from 1–21 with a mean value of 9.84. Quadrivalent associations ranged from 0 to 2 with a mean value of 1.24. The total number of univalents was 105. The total number of chiasmata recorded was 676 with a mean value of 27.04 ranging from 0–49 (Table 2). Out of 676 chiasmata 494 were terminalized and 182 were unterminalized yielding a terminalization coefficient of 0.73.

#### *C. sylvatica*

PMCs analysed showed 32% trivalent associations and the rest showed both bivalent and multivalent associations along with univalents (Fig. 2i–l). The number of bivalents ranged from 0–29 with a mean value of 16.96. The ring bivalent ranges from 0–11 with a mean value of 4.76 and rod bivalents ranged from 0–24 with a mean value of 12.20. Trivalents ranges from 0–21 with a mean value of 8.52. Quadrivalent associations ranged from 0 to 3 with a mean value of 0.56. The total number of univalent was 33. The total number of chiasmata observed was 1365 with a mean value of 48.75 (Table 2). The total number of terminalized chiasmata was 1003 and unterminalized was 362 and yielding a terminalization coefficient of 0.74.

A low frequency of multivalent as compared to bivalent associations was recorded in all the species (Table 3). In group I, the highest percentage of bivalents was recorded in *C. mangga* (94.02%) and lowest in *C. haritha* (64.12%) and the lowest multivalent association was recorded in *C. haritha* (2.11%) and highest in *C. montana* (5.14%). In group II, the highest frequency of bivalents was recorded in *C. sylvatica* (61.99%) and the lowest in *C. longa* (44.31%).

**Table 3.** Percentage of chromosome associations during male meiosis in *Curcuma* species.

	*C. comosa*	*C. haritha*	*C. mangga*	*C. montana*	*C. caesia*	*C. longa*	*C. sylvatica*
**Quadrivalents**	3.13	2.11	2.89	5.14	5.30	4.52	2.05
**Trivalents**	–	–	–	–	31.32	35.86	31.14
**Bivalents**	83.98	64.12	94.02	77.23	53.09	44.31	61.99
**Univalents**	12.89	33.77	3.89	17.63	10.29	15.31	4.82

## Discussion

In the present study, seven species of *Curcuma* showed varying degree of chromosome association(s) viz. bivalents, multivalents and univalents. Group I species showed the prevalence of bivalent associations besides univalents and occasional quadrivalents with a near- normal meiotic behaviour. On the other hand Group II species as expected showed trivalent associations besides bivalents, univalents and quadrivalents. Similar observations were also reported by Ramachandran 1961, Nambiar 1979 and Puangpairote et al. 2016 in *C. aromatica*, *C. decipens*, *C. longa, C. comosa* and *C. latifolia*. It is interesting to note that univalents in *C. montana* lie in close proximity to each other at diplotene suggesting a residual attraction between homologues and their recent separation (Ghosh et al. 2016). However, in the remaining six species, the occurrence of univalents cannot be deciphered whether it is a consequence of synaptic variation or precocious separation of the chromosomes.

The present study strongly support that *Curcuma* is an allopolyploid complex which is evident from the low frequency of multivalent associations and in view of the fact that chromosome associations at the first meiotic division are the usual source of information concerning the type of polyploidy in a given plant (Swaminathan 1953). Allopolyploidization mechanisms involving interspecific and intergeneric hybridization, followed by chromosome doubling for obtaining a stable allopolyploid lineage, plays a pivotal role in the plant evolution (Stebbins 1971, Feldman and Levy 2005, Ozkan and Feldman 2009, De Strome and Mason 2014). Allopolyploids are characterized by a diploid-like meiotic behaviour. Male meiotic events in *Curcuma* species clearly signify that species differentiation is helped by polyploid events and the resultant products are yet to be stabilized in nature.

Members of the zingiber family viz. *Zingiber* and *Mantisia* exhibit varying degree of meiotic irregularities have contributed to reduce fertility and poor seed set (Ramachandran 1969, Sharma et al. 2012). This might be the probable reason for vegetative propagation by means of bulbils and rhizomes (Puangpairote et al. 2016). Likewise, *Curcuma* species have also adopted vegetative mode of propagation which apparently help to overcome meiotic disturbances. Furthermore, polyploidy has offered a strong evolutionary advantage to adapt to a wide range of ecological niche and better survivability than their diploid counterpart (Stebbins 1971, Grant 1971, Feldman and Levy 2005). Several studies have reported that *Curcuma* species with 2n = 63 (probable triploids) are geographically widespread (Ramachandran 1961, Škorničková et al. 2007) and have been slightly successful in cultivation, mainly for their productive rhizomes and competitive ability in natural environment (Puangpairote et al. 2016).

**Figure 3.**
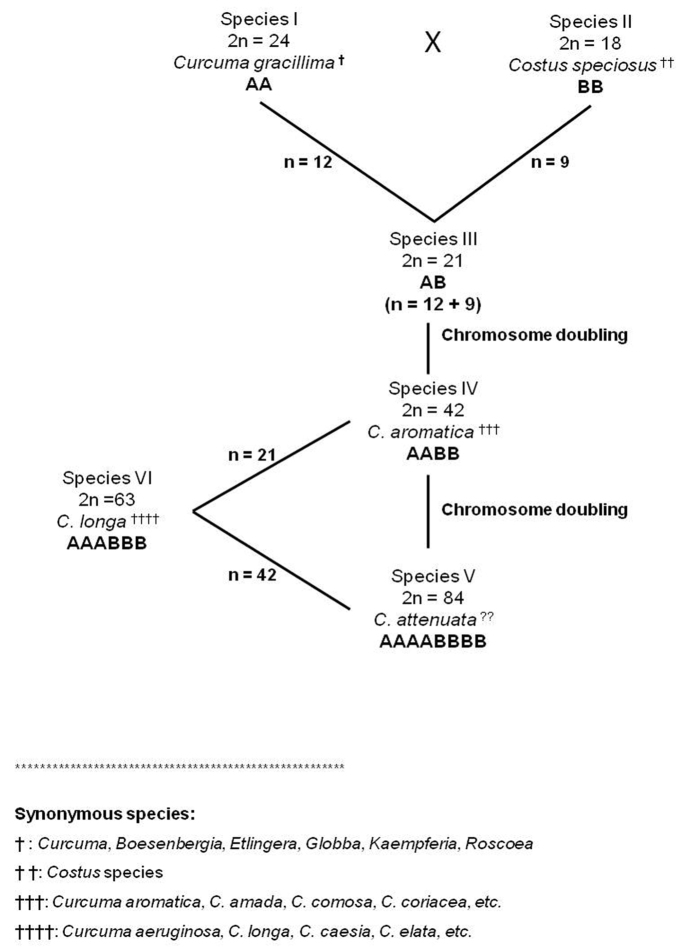
Proposed scheme for *Curcuma* speciation and diversification.

From comprehensive male meiotic investigation in seven species of *Curcuma*, we speculate that the speciation in *Curcuma* might have been affected by inter-specific crosses. We hypothesize that *Curcuma* species with 2n = 24 (e.g. *Curcuma gracillima*, etc.) might involved in hybridization events with species of related taxa belonging to the order Zingiberales having 2n = 18 (e.g. *Costus speciosus*) resulting in F_1_ progeny with 2n = 21 (Fig. 3). Such hybridization events might be followed by natural and expected chromosome doubling giving rise to amphidiploids with 2n = 42, a somatic number more common in the genus *Curcuma* e.g. *C. aromatica*, *C. mangga*, *C. decipens*, etc. In the course of subsequent evolution, these amphidiploid species might have underwent yet another round of chromosome doubling resulting in species derivatives with 2n = 84, a presumed octoploid viz. *Curcuma attenuata*. Few probable triploid species of *Curcuma* such as *C. caesia, C. longa, C. sylvatica*, etc., could be possible due to inter-specific hybridization at heteroploid levels involving amphidiploids (e.g. *C. aromatica*, *C. comosa*, *C. mangga*, etc.) and inter-specific octoploid (e.g. *C. attenuata*). Our hypothesis amply gains support from cytogenetical investigation carried out in the present study, wherein male meiotic analysis of amphidiploid species viz. *C. mangga* showed the presence of more bivalents (94.02%) as compared to univalent or any other type of associations. On the other hand, triploid (presumed) species like *C. longa* showed the presence of significant number of trivalents (35.86%), a hallmark feature of triploids. However, detail meiotic data from species with 2n = 84, like *C. attenuata* (presumed naturally occurring octoploid) needs to be further investigated for approval of the hypothesis proposed.

Besides the reason for considering *Costus speciosus* as a putative diploid parent is that there is no published literature on chromosome counts with 2n = 18 in any of the species belonging to Zingiberaceae, Hedychieae and Globba, the closely related tribes of the order Zingiberales. Moreover, Costaceae showed a close relationship with Zingiberaceae and was even previously placed as a subfamily within the family Zingiberaceae and immensely shared broad similarities in inflorescence and floral traits (Specht and Stevenson 2006). Futhermore, x = 21 is too high a basic number to be considered (Škorničková et al. 2007), therefore, we suggest that the genus *Curcuma* has evolved by hybridization of species with different chromosome numbers of 2n = 24 and 18, resulting in a dibasic amphidiploid species which is in complete support of Ramachandran (1961, 1969) and Nambiar (1979) findings with regard to speciation of the genus *Curcuma*.
